# Significance of clinical observations and biochemical alterations in buffalo calves with dietary abomasal impaction

**DOI:** 10.1186/s12917-017-1325-8

**Published:** 2018-01-02

**Authors:** Maged R. El-Ashker, Mohamed F. Salama, Mohamed E. El-Boshy, Eman A. Abo El-Fadle

**Affiliations:** 1 Department of Internal Medicine and Infectious Diseases, Faculty of Veterinary Medicine, Mansoura University, Mansoura, 35516 Egypt; 2 Department of Biochemistry, Faculty of Veterinary Medicine, Mansoura University, Mansoura, 35516 Egypt; 3 Department of Clinical Pathology, Faculty of Veterinary Medicine, Mansoura University, Mansoura, 35516 Egypt; 4 Department of Laboratory Medicine, Faculty of Applied Medical Science, Umm Al-Qura University, Makkah, 21955 Kingdom of Saudi Arabia; 5 Department of Animal Husbandry and Development of Animal Wealth, Faculty of Veterinary Medicine, Mansoura University, Mansoura, 35516 Egypt

**Keywords:** Abomasum, Alkalosis, Buffalo, Impaction, Oxidative stress

## Abstract

**Background:**

The present study aimed to throw light on the clinical characteristics of abomasal impaction in buffalo calves and its associated biochemical alterations. For this reason, a total of 20 male buffalo calves *(Bubalus bubalis)* with abomasal impaction were studied. The investigated calves were at 6 to 12 months of age and were belonged to three private farms in Dakahlia Governorate besides sporadic cases admitted to the Veterinary Teaching Hospital, Faculty of Veterinary Medicine, Mansoura University, Egypt. Ten apparently healthy buffalo calves were also included as controls. According to the clinical outcome, the diseased calves were categorized into survivors (*n* = 11) and non-survivors (*n* = 9). Blood samples were collected from all animals to estimate blood gases besides a panel of selected biochemical parameters. The definitive diagnosis of dietary abomasal impaction was achieved by either left flank exploratory laparotomy or by necropsy.

**Results:**

Both survivors and non-survivors demonstrated common clinical findings including distension of ventro-lateral aspect of the right abdomen, and varying degrees of dehydration. The great majority of survivors (81%) and 100% of non-survivors were anorexic and had rumen stasis as well as hard texture upon ballottement of the left flank. Approximately 45% of non-survivors had frothy salivation, expiratory grunting and were being tender when strong percussion was applied on the right flank. Diseased calves had metabolic alkalosis, while plasma potassium and chloride were significantly lower in non-survivors than those of survivors (*P* < 0.05). Serum malondialdehyde, superoxide dismutase and uric acid were significantly higher in diseased buffalo than controls and in non-survivors than survivors (*P* < 0.05). Serum total protein, albumin, creatinine, urea, aspartate aminotransferase, gamma-glutamyl transferase, and total bilirubin levels were also higher in non-survivors than those of survivors (*P* < 0.05).

**Conclusion:**

Buffalo calves with dietary abomasal impaction were associated with marked clinical and biochemical alterations that could be helpful for an accurate diagnosis of the disease.

## Background

Abomasal impaction is a rarely reported clinical condition in adult ruminants that occurs due to accumulation of excess solid materials in the abomasum with subsequent enlargement of the organ [1, 2]. Distention of abomasum secondary to a luminal foreign body is more appropriately termed “luminal obstruction” rather than “abomasal impaction” because of its focal nature [3]. It is mainly observed in cows fed on low quality roughage with decreased water consumption [4, 5]. The disease may be observed as a primary disorder whenever straw or similar poor quality fibrous feed constitutes a large portion of the winter diet [6–8]. Other reports have indicated that the disease can be caused by non-food foreign substances, such as sand and gravel [9–11].

Under field conditions particularly in some developing countries, owners feed their feedlot animals a ration composed mostly of poor quality roughage mixed with some grains to reduce the feeding costs and to satisfy beef grading standards [2]. A combination of low digestibility and excessive intake of these roughages could lead to their accumulation in the forestomach and abomasum with a resulting rumeno-abomasal impaction [8]. Other factors that contribute to dietary impactions include high energy demands of growing heifers and cold weather [12]. The disease can also occur as a secondary condition in association with vagal indigestion and traumatic reticuloperitonitis [13, 14]. In the past, vagus nerve damage has been believed to be the key player in the development of abomasal impaction [15, 16]; however, mechanical fixation of the reticulum to the ventral abdominal floor in animals with traumatic reticuloperitonitis interferes with the normal sieving action of the reticulum with subsequent accumulation of fibers in the abomasum leading to its distention [14, 17, 18].

Up to now, there has been limited information about the clinical settings of abomasal impaction in water buffalo and its associated biochemical abnormalities. In line with these considerations, the present study aimed to characterize the clinical findings of abomasal impaction in buffalo calves and to explore the associated biochemical alterations. Here, we hypothesize that abomasal impaction will be associated with characteristic clinical and biochemical alterations that might have diagnostic potential.

## Methods

### Animals and study design

The present study was carried out on a total of 20 male buffalo calves *(Bubalus bubalis)*, belonging to three private farms in Dakahlia Governorate, Egypt (*n* = 18) as well as sporadic cases admitted to the Veterinary Teaching Hospital, Faculty of Veterinary Medicine, Mansoura University, Mansoura, Egypt (*n* = 2) during 2013 and 2014. For comparison, ten apparently healthy buffalo calves from the same population, aged between six to 12 months, were randomly selected and served as a control group.

The main presenting complaints were anorexia for several days, cud-dropping, scanty feces and moderate ventro-lateral distension of the right side of the abdomen. Medical history included feeding on poor quality and coarse roughage consisted of chopped rice straw in alternation with corn silage ad libitum. To obtain a uniform population for this study, data analyses were restricted to buffalo aged between 6 and 12 months, and those who did not receive medication prior to clinical examination.

Dietary abomasal impaction was tentatively diagnosed on the basis of nutritional history, clinical findings, and the results of laboratory investigations, while confirmation was achieved by left flank exploratory laparotomy (*n* = 2) or by necropsy (*n* = 7). The left flank laparo-rumenotomy was performed in standing position under linear infiltrating anesthesia according to the previously described method [19]. According to the clinical outcome, the diseased calves were categorized into survivors (*n* = 11) and non-survivors (*n* = 9).

### Clinical examinations

Thorough physical examinations of diseased as well as clinically healthy calves were carried out according to the standard methods described by Radostits et al. [20]. In brief, a general and close up observations were conducted to evaluate the body condition, demeanor, color of visible mucous membrane, circumference of the abdomen, eating behavior, besides the pattern of urination and defecation. Vital signs including rectal temperature, heart rate, and respiratory rate were essentially recorded. The rectal temperature was measured by using a commercially available mercury thermometer; while the heart rate and respiratory rate were measure by a respective auscutation of the heart and lung. Deep palpation, strong percussion, as well as ballottement were performed on both sides of the abdomen. Tests for reticular foreign bodies were also implemented.

### Sampling and measurements

#### Arterial blood samples

Two mL of blood were collected from all calves via auricular artery into a heparinized syringe and used for measuring blood gases, such as partial pressure of carbon dioxide tension (pCO_2_), partial pressure of oxygen tension (pO_2_), blood pH, and calculating acid base parameters including bicarbonate (HCO_3_^−^) and base excess (BE) using blood gas analyzer (AVL 995-Hb, AVL List GmbH Medizintechnik, AVL Medical Instruments UK Ltd) adjusted to the rectal temperature of the investigated calves. The air bubbles were expelled and the syringes were kept at 4 °C until analysis, which was performed within 30 min. Plasma electrolytes such as sodium (Na^+^), potassium (K^+^), and chloride (Cl^−^) levels were measured by using AVL 984-S Electrolyte Analyzer (AVL List GmbH Medizintechnik, AVL Medical Instruments UK Ltd). The anion gap (AG) was then calculated according to the eq. AG = (Na^+^+K^+^) - (Cl^−^ + HCO_3_^−^), as previously adopted [21]. Blood lactate and glucose levels were also measured spectrophotometrically by using commercial kits supplied by Spinreact (Spinreact, Barcelona, Spain).

#### Venous blood samples

Blood samples were collected from all the examined calves through jugular vein puncture into tubes either containing sodium ethylenediaminetetraacetic acid (EDTA) or plain tubes. The blood samples collected in the EDTA tubes were used for hematological evaluation of packed cell volume (PCV %), total and differential leucocytic counts; while those collected in plain tubes were left to coagulate to separate serum. Only clear non hemolyzed serum samples were collected after centrifugation at 3000 rpm, and kept frozen in aliquots at −20 C^0^ until required for biochemical analyses. Serum samples were used for estimation of calcium, phosphorus, malondialdehyde (MDA), superoxide dismutase (SOD) activity, uric acid (UA), reduced glutathione (GSH), vitamin C (Vit. C), nitric oxide (NO), aspartate amino transferase (AST), gamma-glutamyl transferase (GGT), sorbitol dehydrogenase (SDH), total bilirubin, total protein, albumin, urea, and creatinine concentrations.

Biochemical analyses of the aforementioned parameters were carried out spectrophotometrically by using commercial kits according to the manufacturer’s instructions. Calcium and phosphorus were measured by using kits supplied by Genzyme (Genzyme Diagnostics Co, USA). For MDA, SOD, UA, GSH, Vit. C, and NO measurements, the kits were supplied by Bio Diagnostic (Bio Diagnostic, Cairo, Egypt). For AST, GGT, SDH, and total bilirubin measurements, the kits were supplied by Randox (Randox Laboratories Ltd., UK). For estimation of total protein, albumin, urea and creatinine concentrations, kits were supplied by Spinreact (Spinreact, Barcelona, Spain).

#### Medical management

Besides offering fresh food and water, the following medications were given to affected animals: oral liquid paraffin (El Gomhorea Co. for Chemicals and Pharmaceuticals, Egypt) at a dose of 10 ml/ kg daily for 3 days; a daily I/V infusion of a balanced electrolyte solution (Misr Co. for Chemicals and Pharmaceuticals, Egypt) at a dose of 100 ml/kg for 3 days; the half of that fluid was given in the first 4 h and the remaining fluids were given 3 times daily. To help reestablish the normal rumen flora, a transfaunation was adopted to sick animals. The rumen transfaunate was obtained from a healthy ruminant during abattoir slaughter and was transferred, as soon as possible post-collection, to the recipient animal and was given either at surgery (*n* = 2) or via oroesophageal intubation (*n* = 18), at approximately 8 L as an oral dose. Follow-up information was collected through owner contact, and via referring veterinarians regarding the animal’s health status post treatment.

### Statistical analysis

Data were statistically analyzed using Statistical Package for Social Sciences (SPSS) version 17.0 (USA). Data were tested for normality by using Shapiro–Wilk test, and *P*-values were non-significant; therefore, parametric analysis of variance (general linear model) was used for ANOVA test. The differences between groups were compared by using one-way ANOVA with *post-hoc* Bonferroni multiple comparison test. Least square means ± standard error for each variable were calculated by following formula:

$$ {Y}_{ij}=\mu +{X}_{i1}+{X}_{i2}+{X}_{i3}\dots + Eij $$

Where *μ* is the overall mean of the population and X_i1_, X_i2_, X_i3_ are values of the explanatory (predictor, independent) variables for individual. Y_ij_ is numerical response (outcome, dependent) variable for individual. *E*_*ij*_ is the random error in the observation of the predictors. *P* values at ≤0.05 were considered statistically significant.

## Results

An overview of the detailed clinical settings as well as results of laboratory findings is summarized in Tables 1, 2, 3, 4, 5 and 6. Out of the 20 studied calves, 11 survived, while nine animals perished. Both survivors and non-survivors demonstrated common clinical findings including distension of ventro-lateral aspect of the right abdomen, and varying degrees of dehydration. The great majority of survivors (81%) and 100% of non-survivors were anorexic and had rumen stasis as well as hard mass upon ballottement of the left flank, but rumen fluid splashing sound was evident in approximately 18% among survivors. Scanty feces with hard contents were also evident in 55% of survivors and in 11% among non-survivors. On the other hand, defecation was absent in 45% of survivors and in 89% among non-survivors. Approximately 45% of non-survivors had frothy salivation, expiratory grunting and were being tender when strong percussion was applied on the right flank. Cud-dropping was observed in 33% of non-survivors and in 9% of survivors. The heart rate, respiratory rate and rectal temperature were within the normal reference range in both groups **(**Table 1**)**.

**Table 1:** Clinical findings and outcome of abomasal impaction in buffalo calves (*n* = 20)

Variables	Survivors (*n* = 11)	Non- Survivors (*n* = 9)
Heart rate (Beat/min)	65.72 ± 5.98	66.44 ± 8.24
Respiratory rate (Cycle/min)	14.27 ± 2.14	14.77 ± 1.48
Rectal temperature °*C*	38.31 ± 0.34	38.42 ± 0.35
Appetite	Inappetance (2/11); Anorexia (9/11)	Anorexia (9/9)
Distension of the abdomen	Present (11/11)	Present (9/9)
Defecation	Dry, scanty feces (6/11); Absent (5/11)	Dry and scanty feces (1/9); Absent (8/9)
Dropping cuds	Present (1/11)	Present (3/9)
Frothy salivation	Present (3/11)	Present (4/9)
Expiratory grunting	Absent (11/11)	Present (4/9)
Auscultation of the rumen	Hypomotile (2/11); Stasis (9/11)	Stasis (9/9)
Deep palpation and strong percussion of right flank region	Tender (1/11)	Tender (4/9)
Ballottement of left flank region	Fluid splashing sound (2/11); Firm mass (9/11)	Firm mass (9/9)
Ballottement of the lower portion of the right abdominal flank	Firm mass (11/11)	Firm mass (9/9)

**Table 2:** Least Square Means ± Standard Error of some hematologic variables in buffalo calves with abomasal impaction compared with clinically healthy controls

Variables	Control (*n* = 10)	Survivors (*n* = 11)	Non-Survivors (*n* = 9)
PCV (%)	35.10 ± 0.58^a^	40.09 ± 0.67^b^	45.89 ± 0.68^c^
TLC (10^3^cell/μL)	7.93 ± 0.18	7.49 ± 0.29	7.68 ± 0.37
Neutrophils (10^3^cell/μL)	3.49 ± 0.12	3.31 ± 0.14	3.36 ± 0.14
Band Cells (10^3^cell/μL)	0.06 ± 0.03	0.02 ± 0.02	0.03 ± 0.02
Lymphocytes (10^3^cell/μL)	4.18 ± 0.17	3.91 ± 0.36	3.98 ± 0.47
Monocytes (10^3^cell/μL)	0.18 ± 0.07	0.045 ± 0.02	0.24 ± 0.1
Eosinophils (10^3^cell/μL)	0.02 ± 0.02	0.21 ± 0.1	0.04 ± 0.03

^a, b, c^: Variables with different superscript in the same row are significantly different at *P* < 0.05.* PCV%*packed cell volume; *TLC* total leucocyte count

**Table 3:** Least Square Mean values ± Standard Error of blood gases as well as various acid-base parameters in buffalo calves with abomasal impaction compared with clinically healthy controls

Variables	Control (*n* = 10)	Survivors (*n* = 11)	Non-Survivors (*n* = 9)
pH (mmHg)	7.36 ± 0.001^a^	7.48 ± 0.006^b^	7.56 ± 0.14^c^
PCO_2_ (mmHg)	36.20 ± 0.42^a^	47.27 ± 0.76^b^	51.00 ± 0.69^c^
PO_2_ (mmol/L)	96.60 ± .748^c^	85.18 ± .872^b^	80.78 ± .925^a^
HCO_3_^−^(mmol/L)	24.40 ± 0.34^a^	35.73 ± 0.82^b^	40.44 ± 0.71^c^
BE (mmol/L)	0.20 ± 0.19^a^	10.36 ± 0.73^b^	13.89 ± 0.81^c^
Anion Gap (mmol/L)	16.51 ± 0.49^a^	21.21 ± 0.69^b^	28.36 ± 1.83^c^

^a, b, c^: Variables with different superscript in the same row are significantly different at *P* < 0.05. *PCO*_*2*_ partial pressure of carbon dioxide tension, *PO*_*2*_ partial pressure of oxygen tension, *BE* base excess

**Table 4:** Least Square Means ± Standard Error of serum electrolytes and some metabolic variables in buffalo calves with abomasal impaction compared with clinically healthy controls

Variables	Control (*n* = 10)	Survivors (*n* = 11)	Non-Survivors (*n* = 9)
Sodium (mmol/L)	135.70 ± 0.45	135.55 ± 0.73	136.78 ± 0.465
Potassium (mmol/L)	4.52 ± 0.17^c^	3.00 ± 0.14^b^	2.21 ± 0.01^a^
Chloride (mmol/L)	99.40 ± 0.96^c^	81.73 ± 0.91^b^	70.33 ± 1.8^a^
Lactate (μg/ml)	219.40 ± 2.1^a^	330.45 ± 16.1^b^	690.00 ± 65.6^c^
Glucose (mmol/L)	3.35 ± 0.04^c^	3.00 ± 0.05^b^	2.61 ± 0.11^a^
Calcium (mmol/L)	2.42 ± 0.14^b^	1.93 ± 0.17^a^	1.84 ± 0.15^a^
Phosphorus (mmol/L)	1.27 ± 0.033	1.25 ± 0.030	1.26 ± 0.04

^a, b, c^: Variables with different superscript in the same row are significantly different at *P* < 0.05

**Table 5:** Least Square Means ± Standard error of selected pro-oxidant/antioxidant variables in buffalo calves with abomasal impaction compared with clinically healthy controls

Variables	Control (*n* = 10)	Survivors (*n* = 11)	Non-Survivors (*n* = 9)
MDA (μmol/L)	1.99 ± 0.041^a^	2.71 ± 0.17^b^	3.53 ± 0.115^c^
NO (μmol/L)	4.861 ± 0.078^b^	4.37 ± 0.054^a^	3.14 ± 0.2^a^
SOD (u/ml)	196.12 ± 1.16 ^a^	220.82 ± 1.51 ^b^	249.4 ± 7.8^c^
Vit. C (mg/dl)	3.22 ± 0.01^b^	2.74 ± 0.035^a^	2.59 ± 0.05^a^
GSH (mg/dl)	2.57 ± 0.014^b^	2.40 ± 0.01^a^	2.15 ± 0.04^a^
Uric acid (mg/dl)	1.4 ± 0.01 ^a^	1.71 ± 0.14 ^b^	2.29 ± 0.08 ^c^

^a, b, c^: Variables with different superscript within the same row are significantly different at *P* < 0.05. *MDA* malondialdehyde, *NO* nitric oxide, *SOD* superoxide dismutase, *Vit. C* Vitamin C, *GSH* reduced glutathione

**Table 6:** Least square means ± Standard Error of some liver and kidney function tests in buffalo calves with abomasal impaction compared with clinically healthy controls

Variables	Control (*n* = 10)	Survivors (*n* = 11)	Non-Survivors (*n* = 9)
AST (U/L)	58.05 ± 0.6^a^	68.45 ± 1.23^b^	80.98 ± 4.9^c^
GGT (U/L)	50.90 ± 0.53^a^	60.18 ± 1.24^b^	69.63 ± 3.4^c^
SDH (U/L)	12.07 ± 0.4^a^	27.09 ± 1.41^b^	32.00 ± 4.3^b^
Total bilirubin (μmol/L)	7.27 ± 0.03^a^	7.47 ± 0.024^b^	7.78 ± 0.1^c^
Total protein (g/L)	68.55 ± 0.51^a^	78.90 ± 0.81^b^	84.80 ± 1.69^c^
Albumin (g/L)	39.24 ± 0.30^a^	45.64 ± 0.75^b^	51.13 ± 2.92^c^
Urea (mmol/L)	12.21 ± 0.24^a^	28.27 ± 1.24^b^	36.22 ± 3.55^c^
Creatinine (μmol/L)	61.44 ± 0.67^a^	76.18 ± 1.6^b^	97.62 ± 6.24^c^

^a, b, c^: Variables with different superscript within the same row are significantly different at *P* < 0.05. *AST* aspartate amino transferase, *GGT* gamma-glutamyl transferase, *SDH* Sorbitol dehydrogenase

PCV % was higher in both survivors and non-survivors than controls by 5% and 10%, respectively and was higher in non-survivors compared to survivors. On the other hand, no significant differences were observed in total and differential leucocytic counts between diseased and control buffalo **(**Table 2**)**. Metabolic alkalosis was detected in diseased buffaloes from both groups as indicated by a significant increase (*P* < 0.05) in blood pH, pCO_2_, HCO_3_^−^, and more than 50-fold increase in BE **(**Table 3**)**. Plasma K^+^ and Cl^−^, blood glucose were significantly lower in non-survivors than those of survivors (*P* < 0.05). However, anion gap and blood L-lactate were significantly higher in non-survivors than survivors (*P* < 0.05). Phosphorus and Na^+^ levels did not change among different groups **(**Table 4**)**. Serum MDA, SOD, and UA were significantly higher in diseased buffalo than controls and in non-survivors than survivors (*P* < 0.05). On the other hand, levels of calcium, GSH, Vit. C, and NO were significantly lower in the diseased animals than controls (*P* < 0.05) **(**Table 5**).** Serum total protein, albumin, creatinine, urea, AST, GGT, and total bilirubin levels were also higher in the diseased animals, and were higher in non-survivors than survivors (*P* < 0.05) **(**Table 6**).**

## Discussion

Here, we aimed at studying the clinical and biochemical alterations associated with abomasal impaction in buffalo calves. To date, there has been scarce literature about abomasal impaction in buffalo. In one report, omasal impaction in water buffalo was described [5], and in another one, sand abomasal impaction was reported in two cows [11].

The buffalo calves examined in the present study exhibited common clinical features that are in agreement with previous reports in cows with omasal and abomasal impaction [10, 11, 22]. Interestingly, the non-survivors displayed an expiratory grunting and painful palpation of the right flank region besides dropping of the cuds and regurgitation through the nostrils and the buccal cavity. These signs could possibly be helpful for early prediction of animals with a poor prognosis. In a previous report, it has been stated that cows displaying clinical signs of chronic weight loss, rumen tympany and decreased fecal output are recommended to be evaluated for abomasal impaction [11]. However, in a retrospective study, it has been reported that decreased food intake was considered the most prevailed clinical sign observed in cows with abomasal impaction [2].

In the present study, the increase in PCV% in the diseased calves could be due to dehydration and subsequent concentration of the blood. Similar findings were observed in cows with abomasal displacement [23, 24], and in cows with abomasal impaction [2]. However, in a previous study conducted on cattle and buffalo with omasal impaction, the authors have shown that the majority of the diseased animals demonstrated neutrophilic leukocytosis and lymphopenia and attributed such hematologic alterations to the potential inflammatory complications of impacted feed material [25]. Our results clearly demonstrated that the diseased buffalo suffered from metabolic alkalosis, as evidenced by increased blood pH together with increased blood HCO_3_^−^, and BE. Moreover, apparent hypokalemia, hypochloremia, hyperlactic acidemia, and elevated AG were also observed, and were significantly higher in non-survivors than survivors. In fact, abomasal impaction is considered one of the common causes of alkalosis. It is mainly caused by the continuous secretion of HCl and potassium into the abomasum and inability to evacuate its contents. The observed increase in pCO_2_ could be compensatory due to hypoventilation secondary to alkalosis. Moreover, such an increase in pCO_2_ and a decrease in pO_2_ could lead to anaerobic glucose oxidation and increased lactate levels and AG as observed in our study. Nearly similar findings were previously reported in cows with abomasal impaction [2], and in cattle and buffalo with omasal impaction [25] except that hypochloremia and hypokalemia were not observed in the former study; while, hypochloremia with normal potassium levels were evident in the later report. The hypochloremia observed in the diseased buffalo could be either due to abomasal atony that impairs the flow of HCl into the duodenum or anorexia of the affected animals [8]. The observed hypokalemia could be attributed to the shift of potassium from extracellular to the intracellular space secondary to alkalosis [26]. It has been stated that when abomasal impaction is developed, its contents become drier than normal, with subsequent decline in the rate of emptying. This could lead to hypocalcemia, hypokalemia, and hypochloremia [2].

In the present study, the observed biochemical alterations were in part similar to those reported previously [2, 21, 24]. However, hyperglycemia rather than hypoglycemia was observed [2, 24]. It has been suggested that hypocalcemia could be associated with the development of abomasal impaction as it decreases abomasal motility [27].

Our results demonstrated that abomasal impaction is associated with increased MDA level which is indicative of oxidative stress. The alteration of MDA concentrations was associated with increase SOD activity and decrease in GSH and Vit. C levels. It is well known that free radicals are normally formed from metabolic processes inside the body. Reactive oxygen species (ROS) are derived from oxygen and superoxide anion during mitochondrial electron transport [28]. Accumulation of ROS leads to cellular damage of DNA, proteins, and lipids. There are several antioxidants that scavenge free radicals. Among the enzymatic antioxidants, SOD plays an important role in dismutation of superoxide anions. In addition, there are also non-enzymatic antioxidants, such as GSH and Vit. C [29]. GSH is oxidized with glutathione peroxidase to reduce H_2_O_2_ into two water molecules that requires selenium as a co-factor [30, 31]. Vitamin C is one of the water-soluble vitamins that acts as a direct free radical scavenger [32].

In the present study, the elevated total protein and albumin levels in the diseased buffalo calves could be due to dehydration and increased blood volume. In contrast, other researchers have stated that cows with abomasal impaction showed normal serum total protein concentrations with slightly elevated serum fibrinogen compared with controls [2]. Similarly, in a recent study, it was found that both cattle and buffalo with omasal impaction had normal serum total protein levels with hypoalbuminemia and hyperfibrinogenemia compared with clinically healthy controls [25]. The authors attributed those findings to the chronic starvation or failure of the liver to synthesize adequate amounts of proteins.

The observed elevation in serum creatinine and urea levels could be attributed to a decrease in renal blood flow as a part of compensatory mechanisms to maintain circulation in hypovolemia associated with dehydration, leading to azotemia. Moreover, the significant increase in the levels of liver enzymes in the diseased buffaloes is indicative of liver damage. Such hepatic damage could be due to anorexia and constipation that lead to absorption of toxic substances from the rumen and GIT. The hepatic failure with decreased lactate uptake along with hypoperfusion due to dehydration could also contribute to the observed increase in the blood lactate level [25, 33].

## Conclusion

Buffalo calves with dietary abomasal impaction were associated with marked clinical and biochemical alterations that could aid in an accurate diagnosis of the disease.

## Abbreviations

AG: Anion gap
AST: Aspartate amino transferase
BE: Base excess
Cl^−^: Chloride
GGT: Gamma-glutamyl transferase
GSH: Reduced glutathione
HCO_3_^−^: Bicarbonate
K^+^: Potassium
MDA: Malondialdehyde
Na^+^: Sodium
NO: Nitric oxide
pCO_2_: Partial pressure of carbon dioxide tension
PCV: Packed cell volume
pO_2_: Partial pressure of oxygen tension
SDH: Sorbitol dehydrogenase
SOD: Superoxide dismutase activity
UD: Uric acid
Vit. C: Vitamin C
